# PW03-010 - MHC complexity in Behçet's disease

**DOI:** 10.1186/1546-0096-11-S1-A236

**Published:** 2013-11-08

**Authors:** MJ Ombrello, Y Kirino, P de Bakker, F Cosan, DL Kastner, A Gul, EF Remmers

**Affiliations:** 1 Translational Genetics and Genomics Unit, NIAMS, Bethesda, USA; 2 Department of Internal Medicine and Clinical Immunology, Yokohama City University, Yokohama City, Japan; 3 Division of Genetics, Brigham and Women's Hospital and Harvard Medical School, Boston, USA; 4 Departments of Medical Genetics and of Epidemiology, University Medical Center Utrecht, Utrecht, Netherlands; 5 Istanbul Faculty of Medicine, Istanbul University, Istanbul, Turkey; 6 Inflammatory Disease Section, NHGRI, Bethesda, USA

## Introduction

Family studies support a genetic contribution to Behçet's disease (BD), with a sibling recurrence-risk ratio of 11-52. The class I MHC molecule, HLA-B*51 (B*51), is the strongest known genetic risk factor for BD, however the gene immediately centromeric to *HLA-B*, *MICA*, has also been implicated in BD. Because of strong linkage disequilibrium (LD) between *HLA-B* and *MICA*, their respective contributions to BD susceptibility have been debated. A recent report has proposed that B*51 is not a BD susceptibility allele, and several studies have identified B*51-independent association signals within the MHC.

## Objectives

To clarify the relationship between B*51 and BD, and to test for B*51-independent genetic variation within the MHC that influences BD susceptibility.

## Methods

Using Illumina Human 370CNV SNP genotypes in a Turkish collection of 1244 BD patients and 1303 geographically-matched healthy subjects, we examined SNP haplotypes and LD patterns across the *HLA-B/MICA* region with Haploview. We performed SNP imputation of the MHC using IMPUTE2 and the 1000 Genomes Phase 1 dataset. We inferred classical HLA types and their amino acids using SNP2HLA. Association testing and regression analyses were performed using SNPTEST and SNP & Variation Suite 7.

## Results

We identified a B*51(+) *HLA-B/MICA* haplotype that was strongly associated with BD (p=1.22E-46, OR 2.8). A B*51(-) version of the same haplotype occurred at equal frequencies in cases and controls, demonstrating that B*51 is essential to the risk haplotype. Further, we found that rs2848713, a variant on the *MICA* end of the haplotype, conferred additional risk of BD in B*51(+) individuals. Through imputation, we generated a set of 32,689 imputed SNPs. The 2 most strongly associated SNPs were 4.8Kb centromeric of *HLA-B* (p_min_=1.4E-50), but no SNP was more strongly associated with BD than was B*51 itself (p=1.3E-55). Conditioning on B*51 revealed an association near *HLA-A* (p_min_=5.4E-9), and upon adding a representative *HLA-A* SNP to the regression model, we detected residual association centromeric of *HLA-B* (p=1.5E-5). Analysis of imputed HLA types supported these findings. In addition to the association of BD with B*51 (p=2.2E-55), sequential regression of imputed HLA types identified associations of HLA-A*03 (p=1E-8), HLA-C*0701 (p=9.5E-4), and HLA-B*15 (p=1.2E-4) with BD. Stepwise forward regression of imputed *HLA-B* amino acids identified 6 *HLA-B* residues that together fully accounted for the regional association at *HLA-B.*

## Conclusion

This study affirms B*51 as the strongest risk factor of BD. We have provided strong evidence opposing a B*51-independent role for *MICA* variants in BD susceptibility. We have identified significant effects of HLA-A*03 and HLA-C*0701, which protect against BD, and HLA-B*15, which confers risk of BD. We have identified a group of *HLA-B* amino acids, most of which reside in the antigen binding groove, that together account for the entire association signal at the *HLA-B* locus.

## Disclosure of interest

None declared

